# Global interfertility and heterosis in sugar kelp populations: a next step in sugar kelp breeding

**DOI:** 10.1007/s10811-025-03447-7

**Published:** 2025-01-23

**Authors:** Job Cohen, Robert Twijnstra, Jessica Schiller, Gabriel Montecinos Arismendi, Brigit Reus, Karline Soetaert, Klaas Timmermans

**Affiliations:** 1 Department of Estuarine and Delta Systems, NIOZ Royal Netherlands Institute for Sea Research, 4401NT Yerseke, The Netherlands; 2 Centre for Isotope Research (CIO) – Oceans, Energy and Sustainability Research Institute Groningen, Faculty of Science and Engineering, University of Groningen, PO Box 11103, 9700CC Groningen, The Netherlands; 3 Hortimare, 1704CC Heerhugowaard, The Netherlands

**Keywords:** Seaweed aquaculture, Selective breeding, *Saccharina latissima*, Phaeophyceae, Interregional hybrids, Interfertility, Heterosis

## Abstract

The potential of seaweed aquaculture is restricted by high labor, production and processing costs, leading to low economic viability. Selective breeding can improve yields and cultivation efficiency, thereby decreasing production costs. Until now, genetic resources as input for *Saccharina latissima* breeding trials have been sourced strictly locally, due to concerns regarding outplanting genetically exogenous material in local waters. Here we study, for the first time, worldwide interregional fertility of the seaweed *S. latissima,* in order to assess the potential of including global *S. latissima* genetic resources for selective breeding with regard to heterosis. We quantified the yield (as an indicative aquacultural performance) and morphological traits of intra- and interregional *S. latissima* hybrids originating from a broad range of locations in a common garden experiment. Our results show that the practical application of worldwide *S. latissima* genetic resources in breeding programs is feasible based on global interfertility. We found a wide morphological diversity of hybrids and observed significant heterosis in interregional hybrids. The degree of heterosis could not be linked to geographic distance. These findings reveal that worldwide genetic resources can considerably contribute to *S. latissima* breeding programs and could offer a major next step in improving yields and quality traits.

**Supplementary Information:**

The online version contains supplementary material available at 10.1007/s10811-025-03447-7.

## Introduction

The worldwide demand for high quality seaweed biomass is ever-growing and has gained increasing interest in recent years (Araújo et al. 2021). Farmed seaweeds can serve as a sustainably produced biomass, fitting well within envisioned circular and biobased economies, meanwhile contributing to the UN Sustainable Development Goals (Duarte et al. 2022). Apart from Asia that currently produces more than 99% of the worldwide seaweed biomass (FAO 2022), seaweed aquaculture is an underdeveloped industry. This is often due to high labor, production and processing costs, leading to low economic viability (Van den Burg et al. 2016).

Initiatives for large-scale seaweed cultivation are now emerging around the world. One of the main species of interest for large-scale seaweed cultivation in Europe and North America is the cold-water-adapted brown alga *Saccharina latissima*, also known as sugar kelp (Laminariales). Its relatively well-understood life cycle (Ebbing et al. 2020, 2021b) and ease of cultivation on ropes in a farm setting, coupled with the fast accumulation of biomass and a large yield potential, make it a suitable and attractive crop to farm.

A range of technological improvements could substantially lower biomass production costs and make seaweed aquaculture of greater economic interest (Bak et al. 2018; Barbier et al. 2020; Selnes et al. 2021; Van den Burg et al. 2021). One of the driving forces that could improve yields and cultivation efficiency is selective breeding (Goecke et al. 2020; Huang et al. 2023). Strain development by means of selective breeding for kelps is based upon the diphasic nature of their life cycle, where gametophyte clone cultures can grow vegetatively over prolonged periods and are maintained as a seedstock (Goecke et al. 2020; Wang et al. 2023). Thus far, obtaining higher yields has been a pivotal breeding target in selective breeding efforts for *S.* *latissima* (Umanzor et al. 2021). Improving resistance against (a)biotic stressors or increasing biomass quality are likewise attractive prospects and have been proven to be feasible based on breeding efforts for *Saccaharina japonica* (Hwang et al. 2019; Hu et al. 2021).

Selective breeding programs are founded on interfertility and genetic diversity. Recent breeding efforts for *S. latissima* exclusively utilized gametophytes derived from local sporophytes, due to concerns regarding outplanting genetically exogenous sporophytes in local waters (Campbell et al. 2019; Hwang et al. 2019; Barbier et al. 2020; Guzinski et al. 2020; Mao et al. 2020). These outplanting constraints could, however, be overcome by the creation of sporeless sporophytes (Vissers et al. 2024), which mitigates the risk of introducing foreign genetic material. These sporeless sporophytes could allow unlocking the full potential of the worldwide *S.* *latissima* genetic diversity, which could greatly aid and accelerate breeding efforts and could offer a major advance in kelp breeding (Goecke et al. 2020). Unique gene combinations, alongside increased heterozygosity as a result of using genetically more diverse bioresources, could set the basis for heterosis and increased genetic gain. Heterosis is defined as the superior performance of offspring compared to the performance of its parents (Birchler et al. 2010). Heterosis has been described for various characteristics in *S.* *japonica* (Li et al. 2008, 2016a, b; Zhao et al. 2016) and other kelp species or interspecific hybrids (Westermeier et al. 2010, 2011; Martins et al. 2019; Liesner et al. 2022). In one of these studies, heterosis was correlated to genetic distance (Li et al. 2008), showing the potential of crossing distant populations on a global scale to utilize their genetic diversity.

Understanding the genetic diversity of species is essential to support genetics-based crop improvement and for elucidating the underlying mechanisms of potential heterosis. Recent studies have provided important information about the genetic and phenotypic structuring of *S. latissima* at varying geographic scales, as synthesized by Diehl et al. (2023a). The largest genetic differentiation across *S.* *latissima*’s worldwide distribution is found along its longitudinal range, where different phylogenetic groups have been characterized for the Pacific and West and East Atlantic (Neiva et al. 2018). Latitudinal variations in genetic composition have also been recorded: a split in northern and southern genetic clusters has been observed in the Northwest and Northeast Atlantic phylogroups (Neiva et al. 2018). More regional studies show strong genetic structuring (Guzinski et al. 2016, 2020; Møller Nielsen et al. 2016; Breton et al. 2018; Luttikhuizen et al. 2018; Mooney et al. 2018; Mao et al. 2020; Grant and Chenoweth 2021; Ribeiro et al. 2022; Diehl et al. 2023b). The worldwide genetic patterns and strong genetic structuring are thought to be caused by the inability of spores to travel over long distances and the presence of geographic barriers, like ice sheets during glacial periods, barrier-forming variations in salinity, peninsulas, or long stretches of sandy coastlines (Luttikhuizen et al. 2018; Mao et al. 2020; Ribeiro et al. 2022; Diehl et al. 2023a). In contrast to between-population genetic structuring, generally low levels of genetic variation have been found within populations (Guzinski et al. 2016, 2020; Møller Nielsen et al. 2016; Luttikhuizen et al. 2018; Mao et al. 2020; Grant and Chenoweth 2021).

The general observed genetic diversity is reflected by large variations in morphological diversity at sea (Mao et al. 2020; Diehl et al. 2023b). Nevertheless, morphological expression is a combined effect of not only genetic background, but also environmental conditions. Kelps are known to exhibit large phenotypic plasticity, with their morphology changing in response to varying environmental conditions (Zhu et al. 2021; Diehl et al. 2023b). These changes are thought to be controlled by epigenetic adaptive mechanisms (Scheschonk et al. 2022). This large plasticity may have strong impacts on the accuracy of selective breeding programs through genotype by environment interactions (GxE), leading to a shift in performance of hybrids when grown in different environments (Annicchiarico 2002; Huang et al. 2023) Common garden experiments -experiments where populations from multiple geographic locations are grown together under identical conditions- allow to minimize the impact of fluctuating environmental conditions on morphology and can shed light on the heritability of traits under specific environmental conditions. The performance of hybrids should afterwards be confirmed throughout multiple environments.

Interfertility among global *S. latissima* strains, the aquacultural performance and morphological expression of worldwide genetic resources grown under “common garden” conditions, along with possible interregional heterosis effects, remain understudied. This study is the first to explore worldwide *S. latissima* interregional fertility, with the aim to determine the potential of including worldwide *S. latissima* genetic resources for selective breeding with regard to heterosis. We quantified the yield (as an aquacultural performance metric) and morphological traits of intra- and interregional *S. latissima* hybrids of a range of worldwide locations in a common garden experiment. To represent regional genetic diversity, hybrids were constructed by crossing a representative mix of female gametophytes from one region with a representative mix of male gametophytes from the same or another region. Assuming that genetic diversity increases with geographic distance and provided that *S. latissima* is globally interfertile and hybridization leads to heterosis, we hypothesize that heterosis increases with increasing geographic distance. More specifically, we hypothesize that observed heterosis is more prominent in hybrids along the longitudinal direction of dispersal than the latitudinal direction of dispersal.

## Materials and methods

### Experimental design and gametophyte selection

**Fig. 1:**
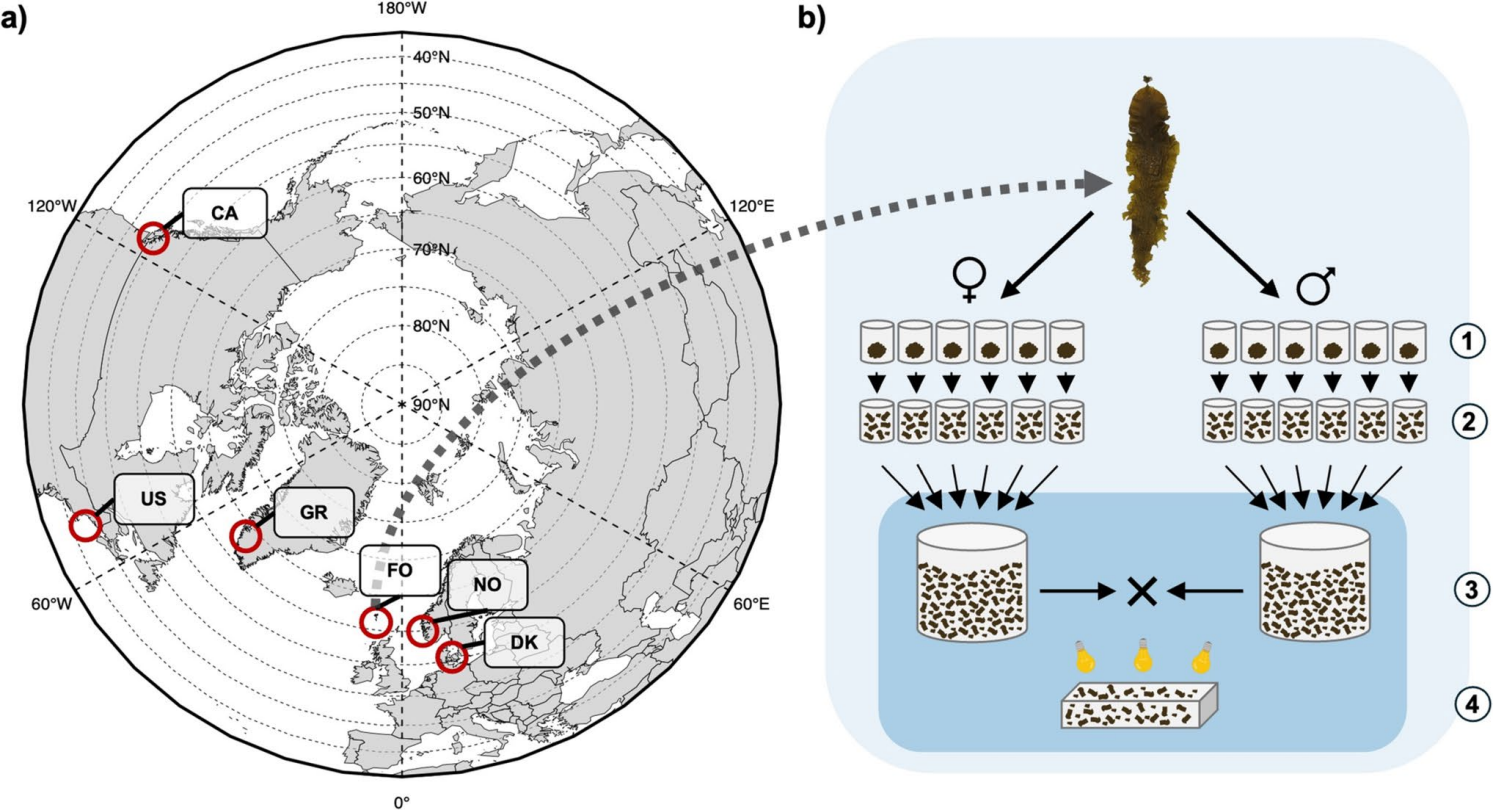
Workflow of the hybridization experiment showing a) the regions of origin of the *Saccharina latissima* genetic resources used to construct the intra- and interregional hybrids and b) the method of constructing the intra- and interregional artificial wildtype hybrids, with material from the Faeroe Islands as illustrated example. Regions are abbreviated according to their country of origin: Canada (CA), United States of America (US), Greenland (GR), Faeroe Islands (FO), Norway (NO), and Denmark (DK). Each region can be comprised of one or multiple sampling locations for sporophyte collection (Table S2). Mature sporulating blades were collected to establish and propagate gametophyte clone cultures (1). Six clone cultures were homogenized (2) and used to construct female or male artificial wildtype pools (3). Crossing females and males from 6 locations, resulted in 36 hybrid combinations (4)

*Saccharina latissima* gametophytes of six different regions (Fig. 1a) were intraregionally and interregionally hybridized, resulting in 36 hybrid combinations (Table S1). A representative mix of six female gametophyte clones from one region and six male gametophyte clones from the same or another region were crossed per hybrid combination (Fig. 1b; Table S2). This was done to represent locally-available genetic diversity and to increase the success rate of a cross. Hybrid combinations are labelled as “LOCATION(f)xLOCATION(m)”, where “f” and “m” represent the female and male gametophyte pool respectively. Clone cultures were chosen based on their parental sporophyte location of collection, gametophyte biomass availability and the culture’s health status. Each gametophyte clone originated from a distinct sporophyte. Following these restrictions, some hybrids contained gametophytes strictly collected from one location within a region, whereas other hybrids contained gametophytes sourced from multiple locations within a region. Exceptionally, 5 clones per mix were used if the collection did not allow the selection of 6 clones.

### Gametophyte genetic material

Uniclonal gametophyte cultures originating from the United States were originally isolated and maintained by both the Yarish Lab at the University of Connecticut (UCONN) and the Lindell Lab at the Woods Hole Oceanographic Institution (WHOI) and were provided to this project via an agreement with the National Center for Marine Algae and Microbiota (NCMA). All other uniclonal gametophyte cultures were provided to this project by Hortimare B.V., The Netherlands. Gametophytes were retrieved from wild sporulating individual sporophytes, collected at varying locations and throughout different years (Table S2). Clonal gametophyte cultures that originated from the United States were constructed according to the culturing protocol mentioned in Umanzor et al. (2021). All other clonal gametophyte cultures were established as described by Ebbing et al. (2021a). Prior to the experiment (one month for the US cultures and two months for the remaining cultures), gametophyte clone cultures were homogenized using Potter–Elvehjem tissue grinders. The cultures were then placed at 10 ℃ under 5 µmol photons m^−2^ s^−1^ red light conditions at a 12:12 h light–dark cycle.

### Crossing, fertilization and seed string construction

One week before inducement of gametogenesis, the gametophyte clone cultures were homogenized again. The gametophyte density for the crossings was set to a dry weight (DW) equivalent of 0.085 mg mL^−1^ by measuring the optical density at 680 nm, which was translated to DW from standard curves. Male and female clones were combined in equal ratios. The inducement took place in flat plastic trays (183 × 135 mm) filled with 100 mL sterile seawater supplied with F medium (Guillard and Ryther 1962) and was set up in duplicate per cross. The prepared trays were placed in red light overnight before being transferred to conditions for gametogenesis inducement (35 μmol photons m^−2^ s^−1^ white light). The trays were relocated every three days to ensure equal light exposure of all trays. After 14 days, 300 μL was sampled in triplicate from the two trays of each cross combined and the sporophyte number per mL induction medium, which was later translated to the number of sporophytes produced per mg female gametophyte biomass, was estimated by counting all sporophytes of two cells or larger. PVC seed string spools (∅ 16 mm, 150 mm long) containing 4.0 m Langman Seaweed Twine (∅ 1.4 mm; Touwfabriek Langman B.V., The Netherlands) were soaked in sterile seawater and covered with sporophytes by spreading the filtered gametophyte/sporophyte mixture from the induction medium over the seed string spools with a paintbrush as described by Li et al. (2022). We targeted for a seeding density of 4000 sporophytes m^−1^ seed string by using corresponding volumes of the induction medium needed to reach this density, which was based on the sporophyte numbers per mL induction medium. The spools were left to dry for 15 min at 10 °C, before being gently transferred to 500-mL PET bottles filled with F/2-medium supplemented sterile seawater. The seed string spools were returned to 35 μmol photons m^−2^ s^−1^ white light conditions at 10 °C. In the week following, the light intensity was gradually increased to 60 µmol photons m^−2^ s^−1^ white light conditions. The bottles were relocated every three days to ensure equal light exposure of all bottles. Every week, the medium was refreshed. After 9 days, gentle aeration was added at the base of the seed string spool through a glass tube. The seedstring spools were left under these conditions for a total of 22 days, before being outplanted. Sporophyte development could be clearly observed 13 days after painting. Some of the hybrids did not show any significant coverage and growth on the seedstring spools and were, therefore, repainted with leftover induction material that was kept at a high density under low irradiance (15 μmol photons m^−2^ s^−1^ white light). All seedstring spools were outplanted on the same date.

### Outplanting and growth conditions

The hybrids were originally outplanted in quadruplicate, at a depth of 40 cm, in two 34 m^3^ tanks (839Lx323Wx128H cm) at the seaweed center of the Royal Netherlands Institute for Sea Research, Texel, on 23 October 2023. Two 316 grade stainless steel cables were spun in parallel over the length of the tank, spaced ~ 0.6 m from either side of the tank, leaving a 2 m space between them for attaching the seeding lines (Fig. S1a,b,c). Eight perforated PVC pipes (spaced 1 m apart) were installed across the width at the bottom of the tank to provide a source of aeration and turbulence. The 144 (36 crosses × 4 replicates) experimental plots (45 cm pieces of seed string containing juvenile sporophytes) were arranged according to a randomized complete block design. Each tank contained two replicates and was divided into two blocks that were generated over the length of the tank, following a slight gradient in bottom aeration. Four experimental plots were stretched over the width of the tank between the steel cables to form one line. In total, there were 18 lines in sequence over the length of the tank, spaced 46 cm apart from one another. Fresh seawater, collected from the Wadden Sea and filtered over a sand-filter, was inflowing at a rate of 250 L h^−1^ per tank. To prevent the spread of any genetically exogenous material, outflowing water was filtered over a sand-filter, collected and UV-sterilized before being released back to the Wadden Sea. Once every 4 weeks, the tanks were emptied and power-washed to remove biofouling and to fully replenish possibly lacking nutrients.

During the 148-day growing period, temperature and light levels were logged at two opposing corners of each tank at 15-min intervals (HOBO Pendant MX Temperature/Light Data Logger, HOBO data loggers, USA). The temperature ranged from 11.6 ℃ at the start of the growing season to −0.5 ℃ during the winter months and increased to 12.0 ℃ at the end of the growing season (Fig. S2). On a weekly basis, nutrient samples were taken for dissolved ammonium, nitrate, nitrite and phosphate quantification (Table S3). As soon as the biomass started to accumulate significantly and macronutrient levels started to drop, a nutrient mix containing 52% NH_4_NO_3_ solution, KH_2_PO_4_ and FeEDDHA 6% (8:1:1 weight ratios) was added. The frequency of application and dosage (Table S4) were based on the algal biomass in the tank, nutrient measurements, previous experience and close monitoring of biofouling. Additionally, salinity measurements were done, showing large variations over time and dropping to as low as 23.8 PSU due to excessive freshwater runoff from rivers during the winter that was characterized by heavy rains (Table S5). All sporophytes in one of the two tanks died during the experiment due to unknown reasons; this left us only with two of the four replicates.

### Harvesting and phenotyping

Harvesting of the lines occurred on 19 March 2024. Each experimental plot was photographed and weighed (drip dry wet weight). The number of blades, with a minimum size threshold of 3 cm in length, was counted and the blade morphological characteristics length, maximum width, surface area, length to width ratio and the angle of the blade base were determined for the 8 longest blades based on photographs using ImageJ (version 1.53t, National Institutes of Health, USA). Lastly, approximately 10 g biomass was frozen at −18 °C, before being freeze-dried to determine the dry weight percentage.

### Further outgrowth to determine mature sporophyte morphology

The longest 8 blades per hybrid were subsequently labeled and outgrown further for 56 days to assess the mature sporophytes’ blade surface structure and frond edge waviness. Outgrowth was done free-floating in 1500 L circular tanks that were center-aerated (causing a tumbling-effect) and temperature controlled (configured to maintain 10℃) to mitigate temperature increases during warm spring days (Fig S1d). The blades were randomly divided over five tanks. The refreshment rate (with sand filtered fresh seawater) of these tanks was 8 L h^−1^ and 10 mL of a nutrient mix containing NaNO_3_ (2.82 mol L^−1^), KH_2_PO_4_ (0.19 mol L^−1^) and FE-EDTA (2.45 mmol L^−1^) was added weekly. To describe the morphology of surface structures, we used the classification and terminology of Zhu et al. (2021). “Bowl: Part of the frond forms raised or depressed areas, similar to a bowl. Spring: Bubbles connected with each other across the center of the frond. Thready: Bubbles connected with each other across the center of the frond. Scattered: Bubbles uniformly distributed along the frond, including the central part.”

### Data analysis

All data analyses were performed using R version 4.1.2 (R Core Team 2021). Plots were generated with R package “ggplot2” (version 3.3.5 (Wickham 2016)). A significance threshold of 0.05 was chosen for all analyses, and the data on plot- and blade-level hybrid performance (*n* = 2) is represented as the mean value ± standard error. For the fertility data (n = 1), two-sided t-tests were performed to find overall significant differences between intra- and interregional hybrids (n = 6 and n = 30 respectively). One-way analyses of variance (ANOVA) were used to examine differences between hybrid groups sharing gametophytes of the same female or male origin (n = 6) to explore any sex-related variations. For the plot- and blade-level hybrid performance, mixed linear models (LM) using package “lme4” (version 1.1–28 (Bates et al. 2015)) were applied to determine significant genotypic differences (n = 2) and differences between hybrid groups sharing gametophytes of the same female or male origin (n = 12). The hybrids and female or male origins were defined as fixed effects and the tank line position as a random effect. The model used for these analyses was: measured variable (e.g. yield or blade density) ~ tested factor (e.g. hybrid/female origin/male origin) + (1|LinePosition). The data were log-transformed to fit normality (0-values were removed before transformation). To determine effects of heterosis in interregional hybrids, the over- or underperformance of traits were estimated over the average trait value of the two corresponding intraregional hybrids: [(F1-MP)/MP] × 100, where F1 is the trait value for the interregional hybrid and MP is the mean trait value of both corresponding intraregional hybrids. If, in any case, one of the two replicates for a hybrid was missing, we removed the other replicate datapoint from the data to still allow the execution of the analysis. Correlation analyses using Pearson correlation coefficients were determined between traits (summarized in a correlation matrix to examine the importance of traits for quality characteristics: wet weight yield and dry weight fraction) and between the degree of heterosis (%) and geographic distance (approximation of the closest distance between locations in km, not crossing land) or the absolute difference in latitude or longitude between locations to discover distance-related heterosis effects.

## Results

### Population-wide global interfertility

**Fig. 2:**
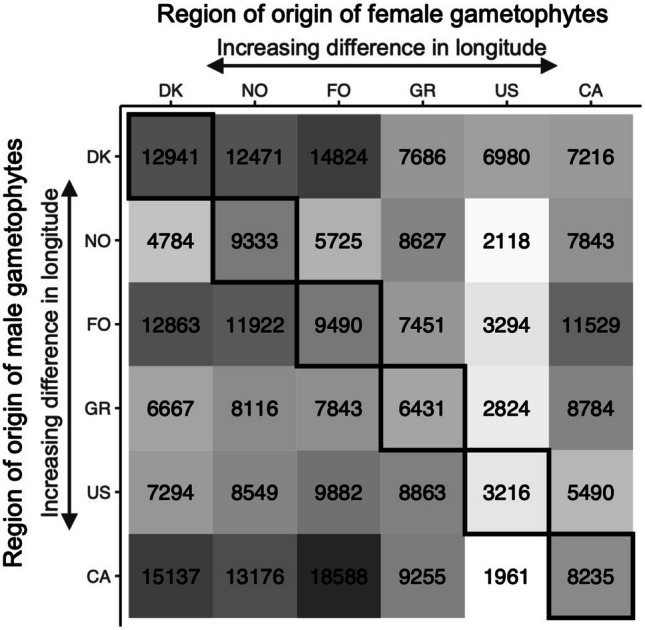
Intra- and interregional hybrid fertility heatmap (number of sporophytes produced mg^−1^ female gametophyte biomass). Intraregional hybrids are found diagonally and are outlined in black. The greyscale reflects the magnitude of the values

All 36 tested hybrids produced seemingly viable juvenile sporophytes (i.e., showing a clear apical-basal patterning and not being identified as parthenogenic sporophytes – as illustrated by Dries et al. (2024)) after the 14th day of induction. Large variations in fertility (number of sporophytes produced mg^−1^ female gametophyte biomass) were found throughout all hybrids (Fig. 2), ranging from 1961 to 18588, with an average of 8539. Comparing intra- and interregional hybrids did not show a significant difference between both groups (two-sided t-test: p = 0.886). Moreover, no significant differences between hybrid groups sharing gametophytes of the same female origin were found (ANOVA: p = 0.111), although a significant difference between hybrid groups sharing gametophytes of the same male origin was found (p = 0.002). This is caused particularly by the low fertility found in the hybrids containing US males, as deduced from a Tukey post-hoc test.

### Intra- and interregional hybrid characterization and performance under common garden conditions

**Fig. 3:**
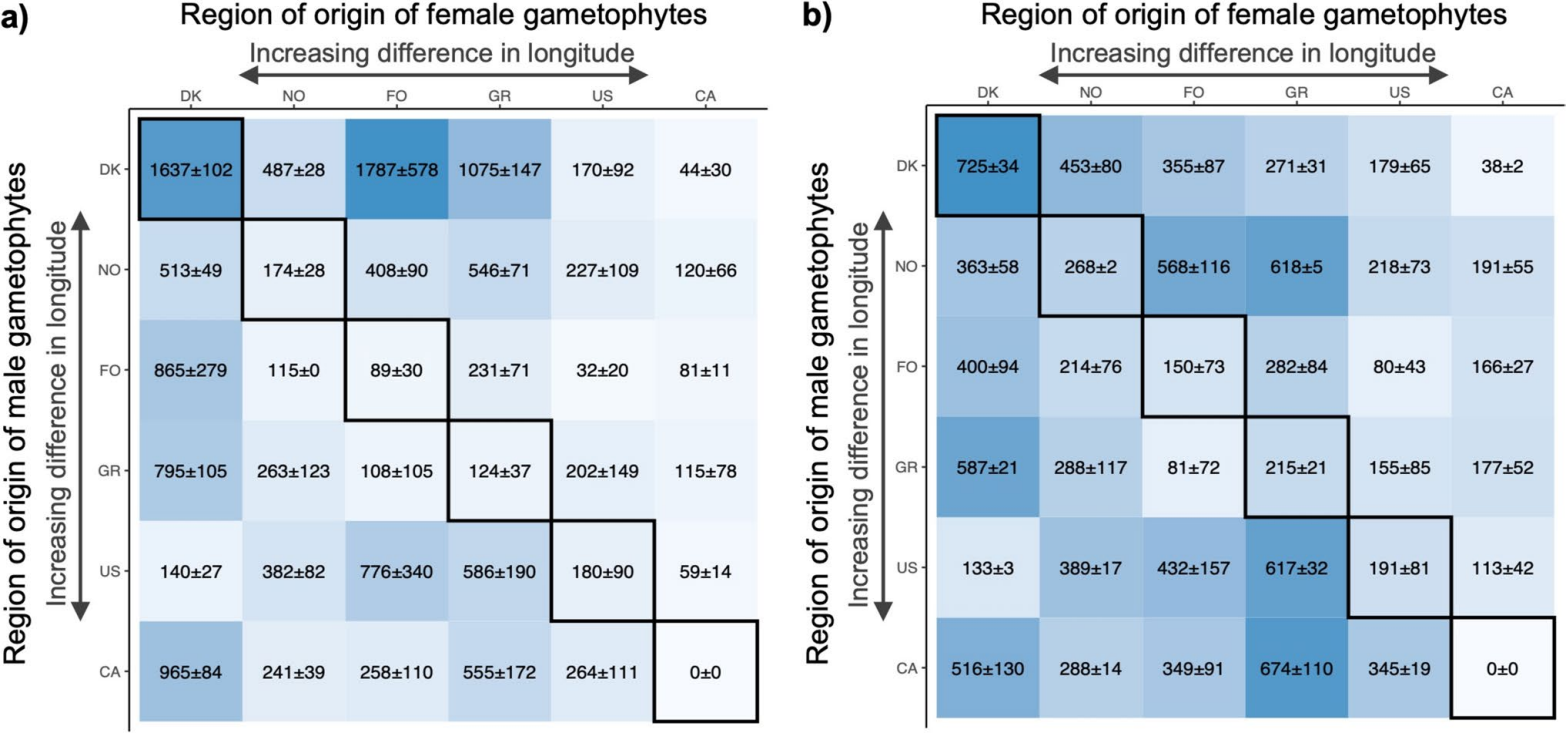
Intra- and interregional hybrid plot data heatmaps showing a) plot wet weight (g m^−1^) ± SE and b) blade density (sporophytes m^−1^) ± SE, both n = 2. Intraregional hybrids are found diagonally and are outlined in black. The color gradient reflects the magnitude of the values

A significant effect of line position (the distance of each line over the length of the tank from one side to the other) on yield (LM: p = 0.007) was found and is explained by the slight imbalance of bottom aeration (and thus water motion) impairing growth under lower water motion. Line position was therefore chosen as a random effect in the mixed linear model used for data analysis. Large overall variations in plot performance (plot weight and blade density) were observed (Fig. 3a; LM: F_34,35,_ = 4.00, p < 0.001 & Fig. 3b; LM: F_34,35,_ = 3.18, p < 0.001). We found no significant difference between plots that originated from seedstring spools that underwent a repainting vs. the original painted spools for both plot weight (LM: F_1,68_ = 0.47, p = 0.495) and blade density (LM: F_1,68_ = 0.24, p = 0.623). The average plot wet weight over all hybrids was 406 g m^−1^ (n = 72). The best overall biomass was obtained in DK(f)xFO(m) with a plot weight of 1787 ± 101 g m^−1^. Among the intraregional hybrids (or artificial wildtype populations), the average plot weight was 369 g m^−1^, with DK(f)xDK(m) having the highest plot weight of 1639 ± 102 g m^−1^. The intraregional hybrid CA(f)xCA(m) did not contain any biomass at harvest and was the worst performer overall. All hybrids containing CA males scored low performance values, due to heavy degradation of their blades. Underwater images from one month prior to harvest showed a normal blade development of these hybrids, although bleaching of the distal ends could already be observed (Fig. S3). The degradation was most likely caused by an unidentified pathogenic infection. This led to a significant effect of male origin group for both plot weight (LM: F_5,64_ = 6.82, p < 0.001) and blade density (LM: F_5,64_ = 5.17, p < 0.001). No significant difference was found in dry weight percentage of the plots (Fig. S4f; LM: F_34,31,_ = 1.36, p = 0.197). The blade density was 308 blades m^−1^ on average for all hybrids, with the highest value reaching 725 ± 34 blades m^−1^ for the intraregional hybrids DK(f)xDK(m). The large variability in blade density counts reflected the observed initial sporophyte coverage on the seedstring spools.

**Fig. 4:**
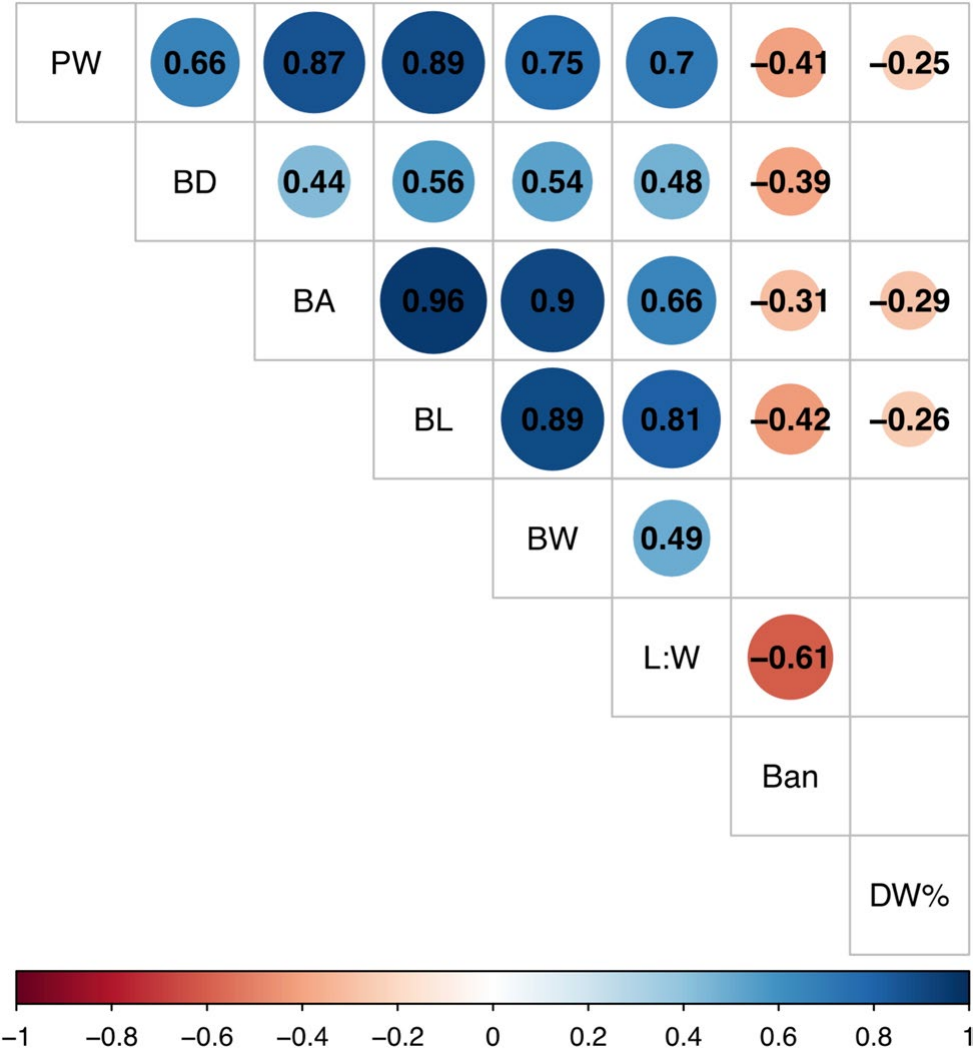
Correlation matrix with pairwise significant (p > 0.05) correlations between measured traits: plot wet weight (PW), blade density (BD), blade surface area (BA), blade length (BL), blade maximum width (BW), blade length: width ratio (L:W), blade angle (Ban), and dry weight percentage (DW%). Numbers and size of the circles indicate the Pearson correlation coefficient between two variables. Insignificant correlations are left blank in the matrix

Large variations in blade level traits (Fig. S4), together with blade density, explain the variation in plot weight. Pairwise trait correlations show significant correlations (p < 0.05) between plot weight and measured variables (Fig. 4). The strongest positive correlations were found between the blade morphological traits blade length, blade width and blade surface area. Plot density also correlated positively with plot weight, although to a lesser extent. Slight negative correlations were found with blade angle and dry weight percentage. Besides these plot- and blade-level trait correlations, additional correlations were found between fertility and plot weight (r = 0.44, p = 0.009), blade length (r = 0.34, p = 0.044), blade width (r = 0.39, p = 0.022) and blade area (r = 0.36, p = 0.033).

### Expression of typical morphological features

**Fig. 5:**
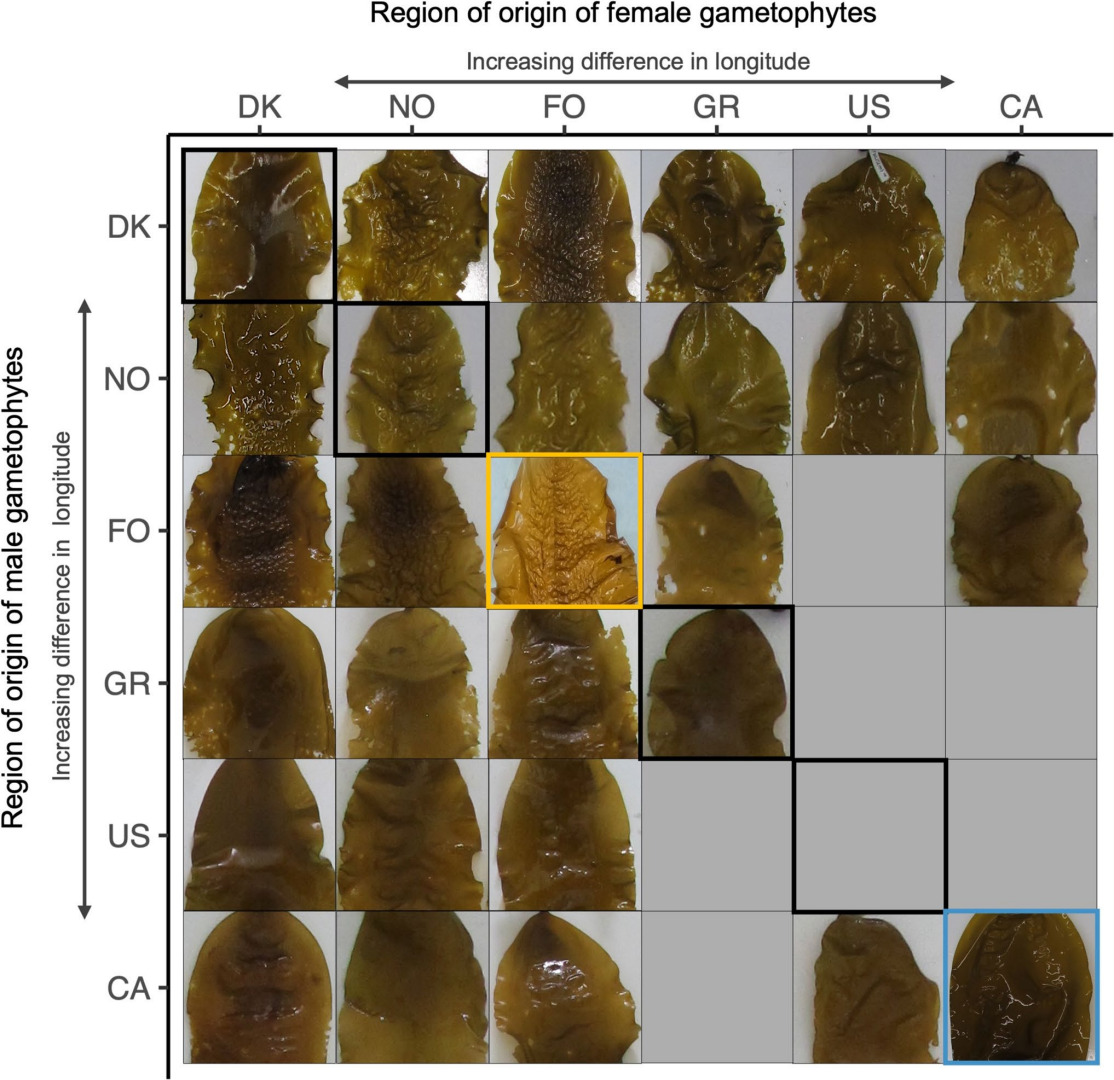
Morphological appearance of intra- and interregional hybrids. One representative sporophyte is displayed as an example for each hybrid. Note that the blade size of the sporophytes varies considerably, and they are scaled to fit the squares. In case sporophytes had degraded or were too small to express any typical morphological characteristics, they were left out of the matrix. Interregional hybrids are found diagonally and are outlined in black. To fill some of the gaps, the sporophyte outlined in blue was photographed from a similar, parallel experiment containing the same starting material, where sporophytes had been outgrown free-floating during their whole lifespan. The sporophytes outlined in yellow is a wild-harvested sporophyte and thus displays its typical wildtype morphology under local environmental conditions

Despite being grown under identical growing conditions in a “common garden” tank setting, the tested hybrids exhibited great differences in morphological appearance: besides variations in length: width ratio (Fig. S4d) and the angle at the blade base (Fig. S4e), a variety of surface structures and variations in frond edge waviness were found (Fig. 5). The strong interpopulation morphological differentiation, as typically seen in wild mature blades (Diehl et al. 2023a), appeared at later growth stages only and became visible mostly after further outgrowth following harvest. Due to the continuation of the previously mentioned (or initiation of a new) pathogenic infection originating from the crosses with CA males, many of the blades deteriorated over time, leading to an incomplete overview. Interestingly, however, susceptibility to the disease seemed to differ for different hybrids.

We found a clear influence of geographical origin on morphological appearance. Interregional hybrids mostly represent the morphological characteristics of (one of) their corresponding intraregional hybrids (Fig. 5). We generally observed that patterned surface structures were dominantly inherited over smooth surface structures, and bowl-/spring-like surface structures were seemingly dominantly inherited over thready-/scattered surface structures. We did not find any clear sex-related dependence on morphological expression.

The DK(f)xDK(m) intraregional hybrid had a smooth surface structure (although some bubble-like structures were occasionally noted within this hybrid too), whereas some interregional hybrids (e.g. DK(f)xGR(m) and CA(f)xDK(m)) showed a more bowl-/spring-like surface structure. A thready-like surface structure was, amongst others, observed in the NO(f)xNO(m) intraregional hybrids and both of its reciprocal crosses with DK gametophytes. The clearest and most apparent surface structure was found for the reciprocal crosses of DK and FO (Fig. 5) that exhibited a scattered, almost bubble-wrap-like surface structure. Although blades of the intraregional hybrid FO(f)xFO(m) degraded over time and are, thus, not shown, this bubble-wrap-like surface structure is typically seen in its wildtype population. We, furthermore, observed that hybrids containing NO gametophytes had a wavier, almost dentate-like frond edge, whereas the DK intraregional hybrid displayed a very smooth and subtle waviness of its frond edge. Hybrids containing US gametophytes exhibited the largest within-hybrid morphological variation.

### Over- and underperformance of blade morphological traits in the interregional hybrids

**Fig. 6:**
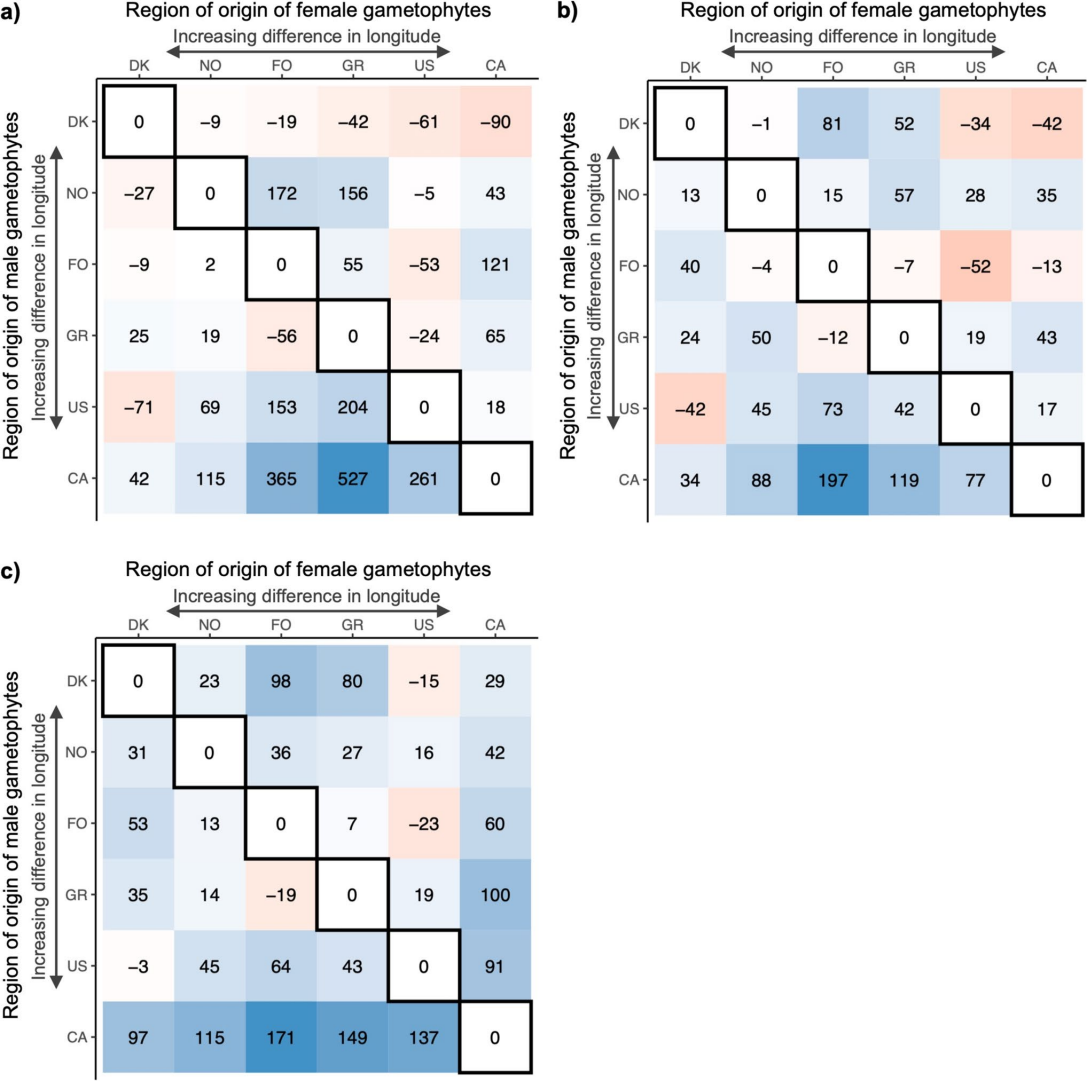
Heterosis values of interregional hybrids (relative over- or underperformance compared to the average performance of corresponding intraregional hybrids) for a) blade length, b) blade width, and c) blade density. Intraregional hybrids are found diagonally outlined in black and received a 0-value per definition

Out of the 30 interregional hybrids, 18 hybrids outperformed their corresponding intraregional hybrids for plot weight, 21 for blade length and 26 for blade width (Fig. 6). Intraregional hybrids, by definition, received a heterosis value of 0%. When comparing the mean heterosis values and their standard errors of the interregional hybrids to base value 0, we identified that the interregional hybrids, on average, significantly overperformed their intraregional hybrids (blade density: 64.97% ± 24.98, blade length: 31.43% ± 9.41, blade width: 51.23% ± 9.12).

These values were subsequently tested for their correlation to geographic distance and absolute distance between longitude and latitude of matching gametophyte locations. This did not result in any significant correlations, except for a significant correlation between geographic distance and heterosis for blade width (Pearson correlation: r = 0.571, p = 0.001), caused by the relatively high heterosis values for CA-originating hybrids due to the dropout of the CA intraregional hybrids (the value 0 was assigned to the intraregional hybrid to calculate the heterosis values).

## Discussion

Selective breeding can support the seaweed aquaculture industry by developing high-yielding cultivars with superior quality characteristics (Goecke et al. 2020). This study is the first to explore the potential of including worldwide *S. latissima* genetic resources in breeding programs and to examine global interfertility allowing hybridization. We found that the genetic material used in our study was globally interfertile. In our common garden experiment, we observed large variations in the hybrid’s aquacultural performance and morphological traits among hybrids. Together with observed population-wide signs of heterosis, we argue that the inclusion of global genetic resources could considerably contribute to *S.* *latissima* breeding programs.

### Global interfertility allows worldwide hybridization

Evolutionary divergence within species may result in hybrid incompatibility or sterility (Ouyang et al. 2010). Despite the appearance of multiple phylogroups that are subjected to incipient speciation and the associated geographic reproductive isolation (Neiva et al. 2018), our results show that the *S. latissima* genetic resources used in this study are globally interfertile. This expands on an earlier crossing experiment, in which crosses between East Atlantic and Pacific *S. latissima* produced fertile hybrids (Bolton et al. 1983), as well as presumable hybridization events between phylogroups detected in the Canadian Arctic (Luttikhuizen et al. 2018; Neiva et al. 2018), and even numerous interspecific *Saccharina* hybridization examples in Asia (Hwang et al. 2019; Hu et al. 2021). Although not covering previously categorized *Saccharina cichorioides*, which was found to form a separate phylogroup in the *S. latissima* species complex, the large geographic range of the *S. latissima* genetic resources used in this study suggests that interfertility extends across its global distributional range. This enables the practical use of worldwide genetic resources for breeding purposes.

Nevertheless, striking differences in fertility were observed between hybrids. These differences in fertility could not be explained by any significant differences in fertility between intraregional and interregional hybrid groups, aligning with findings from fertility experiments with *Macrocystis pyrifera* (Camus et al. 2021). Neither did we find any clear sex-linked effect on fertility. The only significant sex-linked effect identified was the low fertility of US male gametophytes, possibly explained by experienced stress during transport and a decrease in pigmentation observed upon arrival. This finding contradicts a previous report which states that reproductive ability was mainly influenced by the female gametophyte (Song et al. 2024) Variability in fertility and developmental rate of oogenesis and embryogenesis are a common phenomenon found under genetically distinct hybrids (Song et al. 2024) In our study, the differences in fertility within hybrid groups sharing the same female or male gametophyte pool may be explained by specific incompatibility of individual gametophytes used in the artificial hybrid pool, or delays or dissimilarities in reproductive timing due to deviant responses to life cycle controls or genetically imprinted variations in developmental stages for the different locations. Even when the performance of a hybrid sporophyte proves to be optimal for aquaculture purposes, unreproductive gametophytes may raise practical issues for their application in large-scale seaweed aquaculture. Large amounts of gametophyte biomass would need to be cultivated to supply sufficient juvenile sporophytes for cultivation at commercial scale (Ebbing et al. 2021a), resulting in increased production costs (Coleman et al. 2022). Hybrid fertility may, hence, be an important breeding selection criterion that has been undervalued thus far.

### Morphological variety to serve as a basis for breeding high-yielding cultivars

Despite having been grown under identical growing conditions, the tested hybrids showed large variations in plot-level characteristics and blade morphometrics. We opted for an approach to seed with a set number of sporophytes m^−1^ of seedstring to compensate for the earlier mentioned variations in fertility. Yet, initial seedstring spool coverage at the outplanting date and final blade density varied widely. We argue that certain hybrids could have experienced delayed gametogenesis and/or embryonic development and could still have developed sporophytes after having been seeded on seedstring spools, skewing the seeding density of 4000 sporophytes m^−1^ seedstring. This could go hand in hand with possible genetic variability in attachment characteristics.

Multiple traits explain the variations in plot weight that were observed. Blade length seems to be the most important determining factor influencing wet weight yields. Both blade width and blade density were additional important factors that contributed to high yields. These findings partly align with earlier breeding trials (Umanzor et al. 2021; Li et al. 2022), although blade width was not found to significantly positively correlate to wet weight in these studies. We found an unexpected positive correlation between blade density and blade width. Based on earlier trials that showed a slightly negative correlation between these two (Umanzor et al. 2021; Li et al. 2022), we expected a trade-off between, and optimum for, blade density and blade width. A good example of this trade-off is the hybrid DK(f)xDK(m) that had the highest blade density with the highest length to width ratio. The DK(f)xFO(m) hybrid had a much lower blade density but wider blades, and its plot weight was found to be even higher.

Blade shape varied and distinct morphological expression of blade surface structures were observed in our common garden experiment. In most cases, blade surface structure for both reciprocal crosses was shared with one of its corresponding artificial wildtype hybrids. Despite well-known plasticity in morphology in response to changing abiotic conditions (Zhu et al. 2021), our results indicate that location-specific blade morphology (Diehl et al. 2023b) is strongly genetically imprinted. The DK artificial wildtype showed a narrow and long blade morphology with the highest length to width ratio. When hybridized with material from all other regions, a decrease in length to width ratio was observed. Li et al. (2022) found higher yields in hybrids between sugar kelp (*S. latissima*) and skinny kelp (*Saccharina angustissima*); a genetically similar, but morphologically long and narrow species. Although the DK artificial wildtype was high-yielding, DK(f)xFO(m) showed the highest yield overall. This supports the evidence that hybridizing contrasting morphotypes could offer yield enhancements. Offering a wider source of genetic and phenotypic input compared to regional genetic resources only, global genetic resources could benefit *S. latissima* breeding programs (Valero et al. 2017; Goecke et al. 2020).

### Observed population-wide heterosis can support *S. latissima* breeding

This study’s ultimately goal was to investigate heterosis in *S. latissima* interregional hybrids on a global distributional scale, which could further highlight the potential of using worldwide genetic resources. In our study, we examined signs of heterosis based on morphological characteristics such as blade density and blade length and width. On average, the interregional hybrids significantly outperformed the corresponding intraregional hybrids. Exceptions still occurred, although the degree of underperformance never reached the same magnitude as potential overperformance. These findings provide evidence for population-wide heterosis occurring when *S. latissima* genetic resources are crossed interregionally.

Overall, heterosis was not linked to geographic distance. Our hypothesis of stronger heterosis indications along the longitudinal distributional range of *S. latissima* was therefore rejected. This contradicts earlier findings from Li et al (2008) that showed a correlation of heterosis with genetic distance for multiple tested *Saccharina* hybrids. The lacking correlation in our study could be caused by the fact that geographic distance does not directly translate to genetic distance for *S. latissima*. Due to the strong genetic differentiation found between *S. latissima* populations, genetic differences between the populations used could already lead to sufficient heterozygosity needed for heterosis. Another explanation for this could lie in outbreeding depression, which is the reduced performance of hybrids of genetically diverged and locally adapted populations, as discussed by Liesner et al. (2022). Although a mitigating effect on heterosis might have occurred, we could, nevertheless, not find any clear direct indications of reduced performance in distantly related hybrids. Next steps to supplement this work could lie in creating a fine-scale genetic landscape of the material used and could elucidate the genetic basis of the observed heterosis effects. Sequencing data could additionally be used for the implementation of these results in genetics-supported breeding methods, like the use of genomic selection models (Huang et al. 2023).

### Adaptations to abiotic effects as a breeding target or obstacle

This experiment took place in a common garden setting, in a semi-closed-off tank system with limited fresh seawater supply under prevailing ambient environmental conditions. These conditions might have strongly favored the performance of certain hybrids over others, due to embedded location-specific abiotic adaptivity. This may especially explain the variation in performance of the different artificial wildtype populations, e.g., the relatively good performance of hybrids produced with Danish gametophytes. Danish genetic resources used in this experiment originated from locations that are regarded as brackish (Møller Nielsen et al. 2016), with salinity values in the same range as during a large part of our experiment. Alternatively, the microbial community in our experiment may have conceivably benefitted hybrids from nearby origin. Strong variations in performance under different environmental conditions, also known as genotype x environment interactions, heavily impact the accuracy of breeding trials, as also shown for *S. latissima* (Huang et al. 2023). Although our study broadly highlights heterosis in interregional hybrids, direct translation of hybrid performance scores to other environmental conditions should be done with care as they may be highly conditional. This is especially in the interest of developing cultivars for offshore aquaculture, where hydrodynamics and nutrient levels could differ vastly from near-shore aquaculture (Tullberg et al. 2022). A solid understanding of *S. latissima* physiology in relation to (a)biotic conditions is needed to provide better recommendations on breeding strategies.

In conclusion, our results show that using worldwide *S. latissima* genetic resources in breeding programs is feasible based on global interfertility. Understanding the foundation of variations in fertility is essential when upscaling the use of hybrids to large-scale aquaculture. Importantly, developing sporeless sporophytes or other methods of infertility could provide an avenue to overcome outplanting restraints and to enable the deployment of hybrids containing exogenous genetic material in local waters, while at the same time preserving the local genetic diversity. The wide morphological diversity of hybrids and observed heterosis in interregional hybrids suggest that worldwide genetic resources can considerably contribute to *S. latissima* breeding programs and can improve yields and quality traits. The degree of heterosis could, however, not be linked to any geographic distance indicators. Despite the general signs of heterosis in the tested interregional hybrids, large variations in aquacultural performance of the artificial wildtypes indicate potential genetic adaptations to (a)biotic conditions. A solid understanding of *S.* *latissima* physiology in relation to (a)biotic conditions and the continuation of our experimental outcomes throughout different years and at different locations are needed to quantify GxE interactions and provide better recommendations on breeding strategies and hybrid use.

## Supplementary Information

Below is the link to the electronic supplementary material.

Supplementary file1 (DOCX 30831 KB)

Supplementary file2 (XLSX 24 KB)

## Data Availability

Raw data and photos obtained during the experiment are available upon request.
