# Supplementary material for: Global interfertility and heterosis in sugar kelp populations: a next step in sugar kelp breeding
Source: J Appl Phycol. 2025 Jan 23;37(2):1213–26. doi: 10.1007/s10811-025-03447-7 (PMC12055653; doi:10.1007/s10811-025-03447-7)
Supplement: Supplementary file 1 — Supplementary file1 (DOCX 30831 KB) [file 10811_2025_3447_MOESM1_ESM.docx]

Journal of Applied Phycology

Supplementary figures for

"**Global interfertility and heterosis in sugar kelp populations:**

**a next step in sugar kelp breeding**”

Job Cohen^1,2^*, Robert Twijnstra^1^, Jessica Schiller^3^, Gabriel Montecinos Arismendi^3^, Brigit Reus^3^, Karline Soetaert^1^, Klaas Timmermans^1^

^1^ Department of Estuarine and Delta Systems, NIOZ Royal Netherlands Institute for Sea Research, 4401NT Yerseke, The Netherlands

^2^ Centre for Isotope Research (CIO) – Oceans, Energy and Sustainability Research Institute Groningen, Faculty of Science and Engineering, University of Groningen, PO Box 11103, 9700CC Groningen, The Netherlands

^3^ Hortimare, 1704CC Heerhugowaard, The Netherlands

*Corresponding author: Job Cohen (job.cohen@nioz.nl)


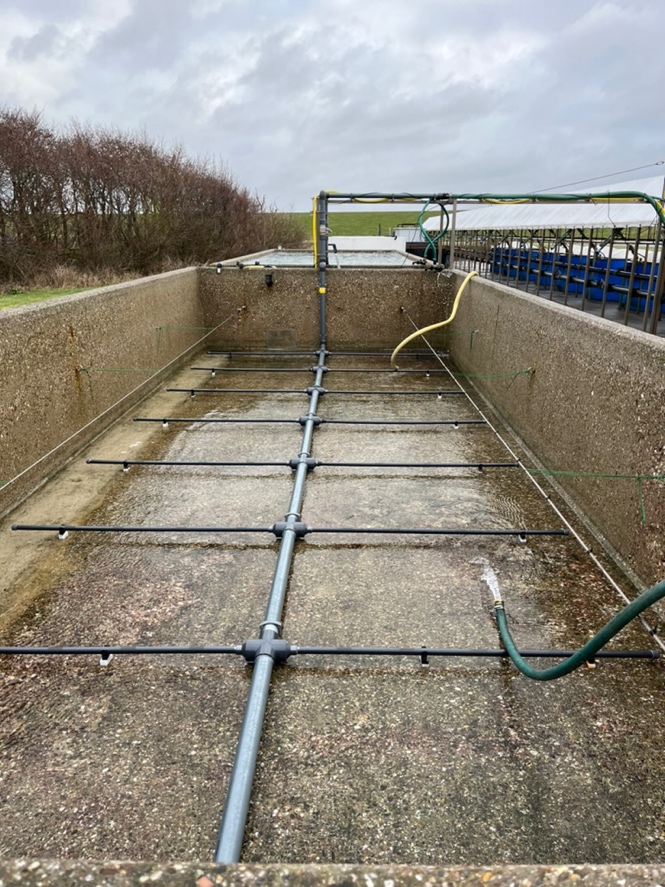

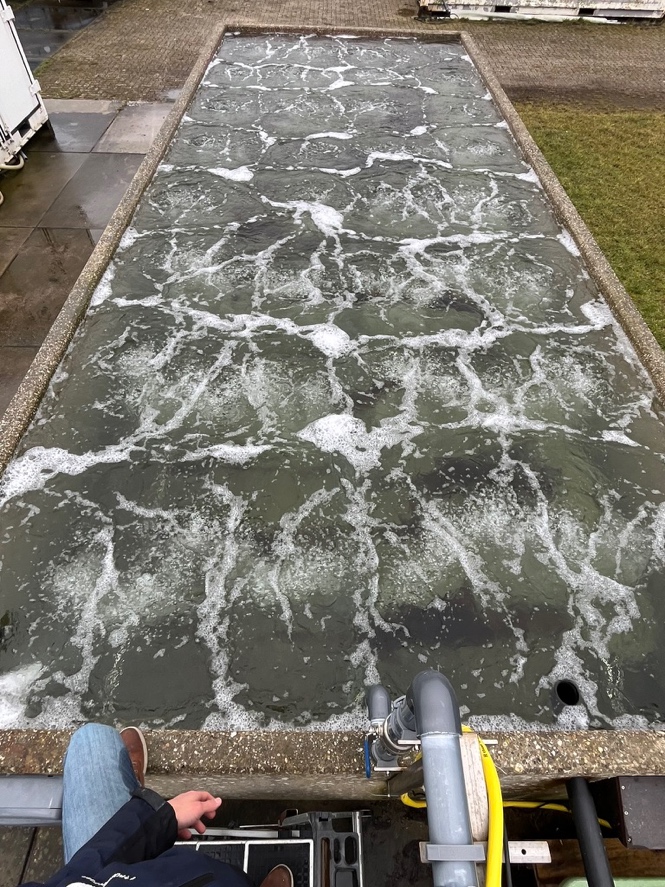

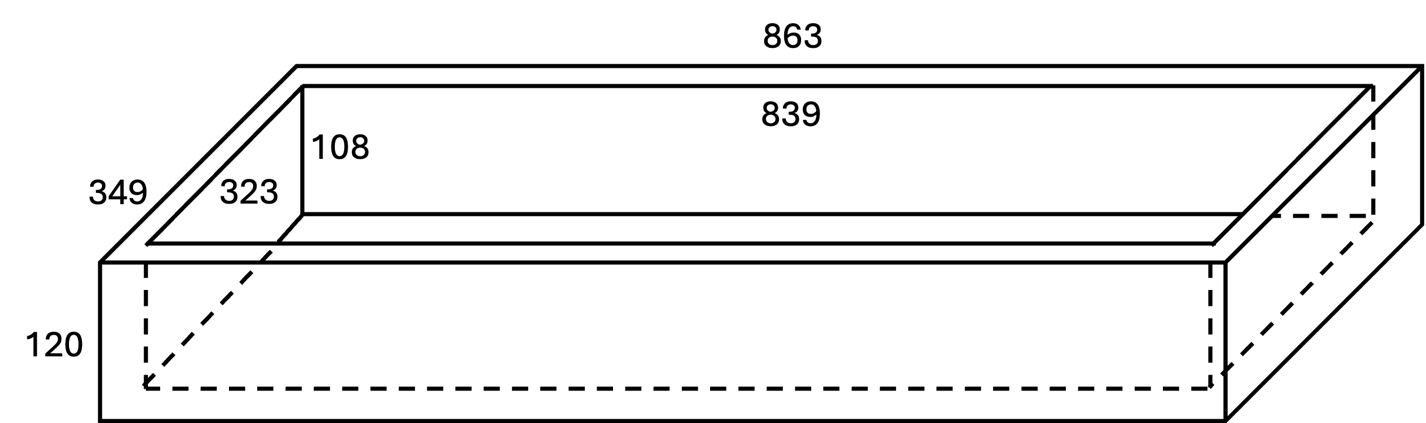

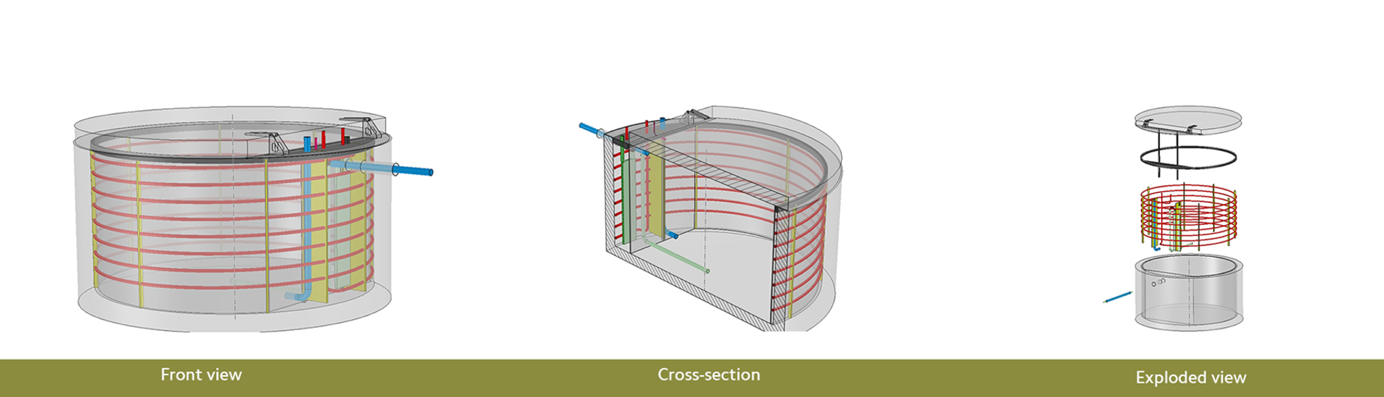


**Fig. S1** Experimental tank setup of the large tank: a) Schematic overview including dimension (in cm), b) cable and bottom aeration placement, c) operating tank experiment with turned-on aeration, and d) specifics of the 1500L circular tanks

d

a

c

b

b

Temperature (℃)

**Fig. S2** Tank water temperature during the experiment


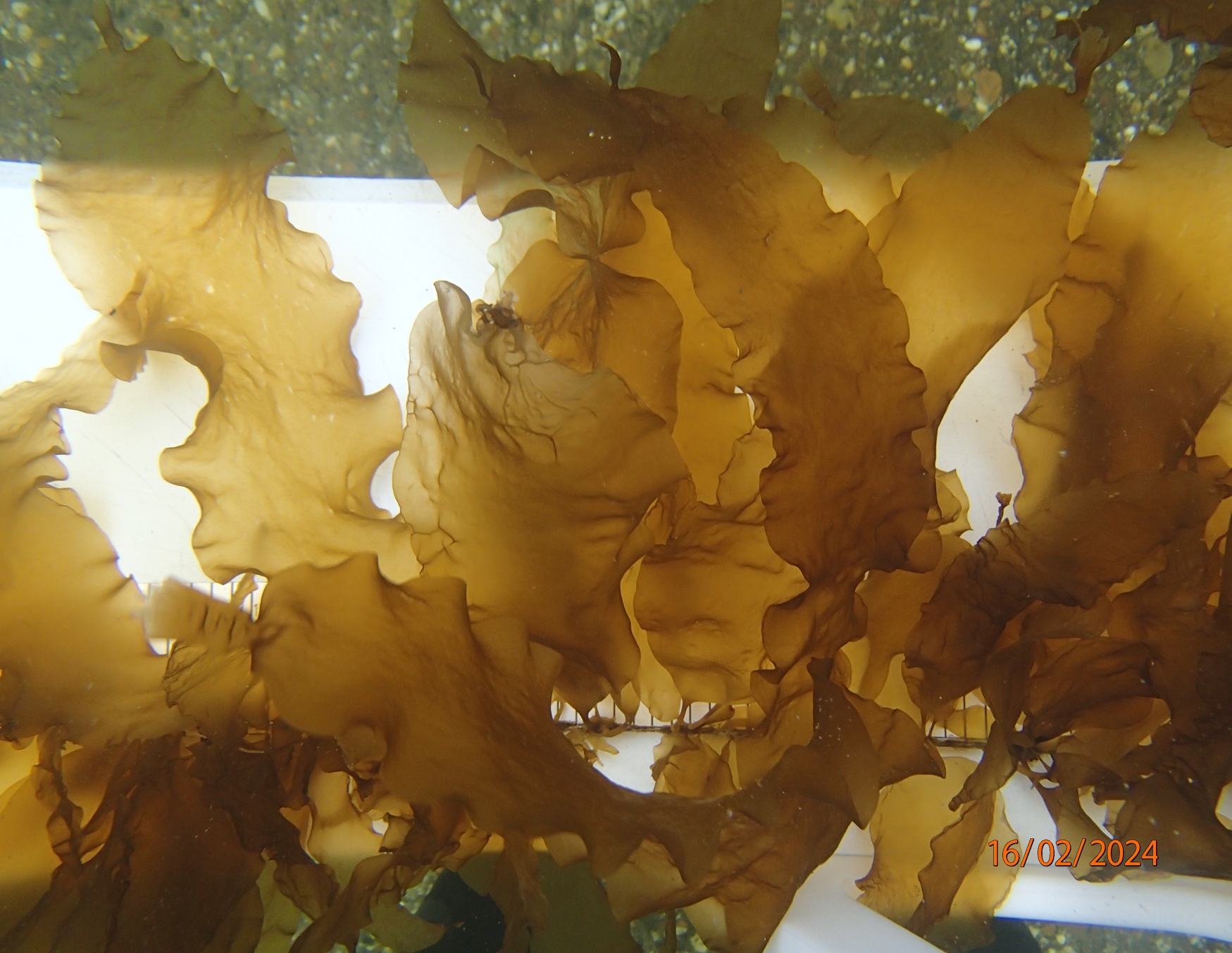

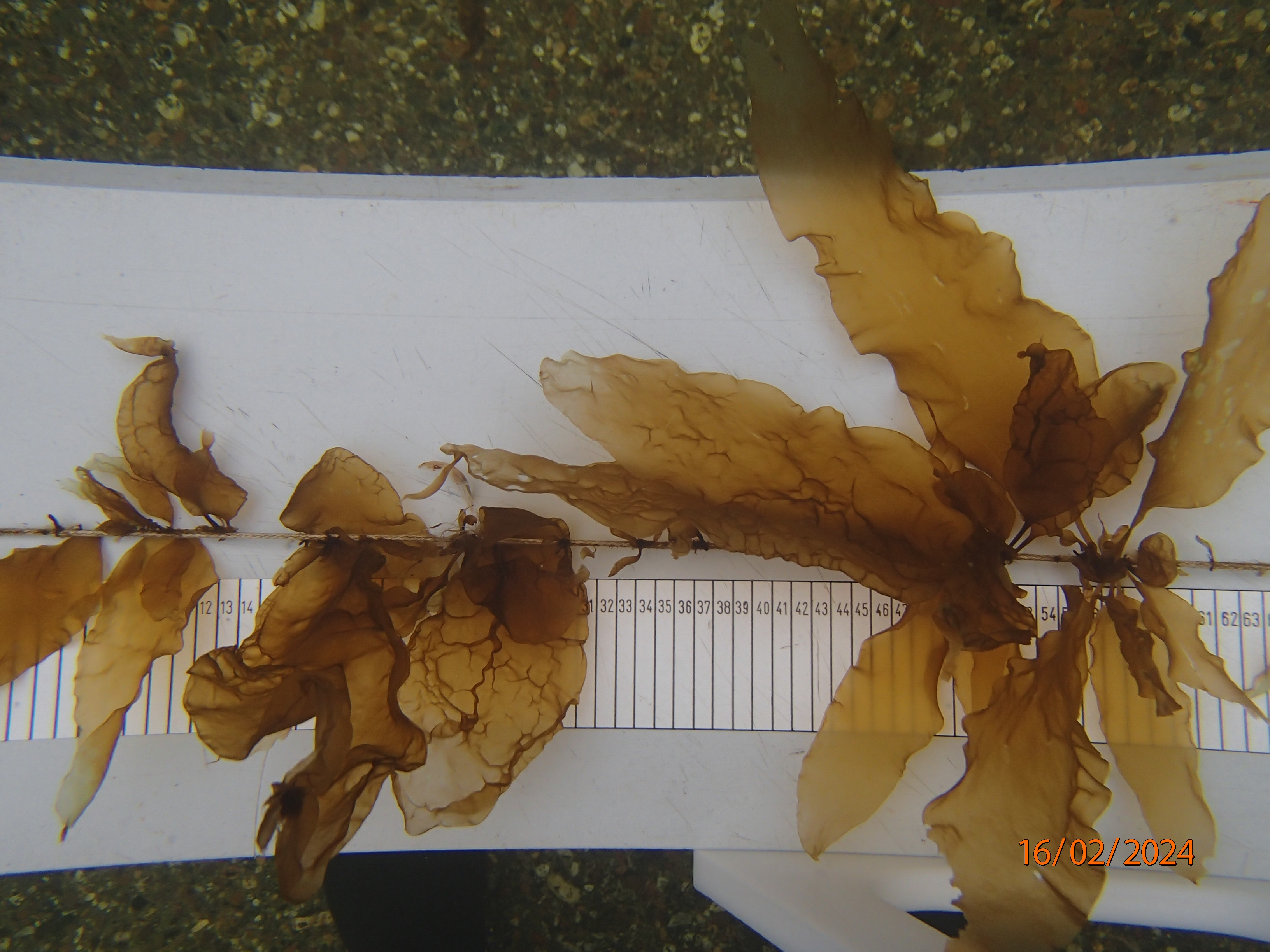


a

b

**Fig. S3** Examples of hybrids containing CA males that show good growth but bleaching tips one month prior to harvest: a) CA(f)xCA(m) and b) GR(f)xCA(m)


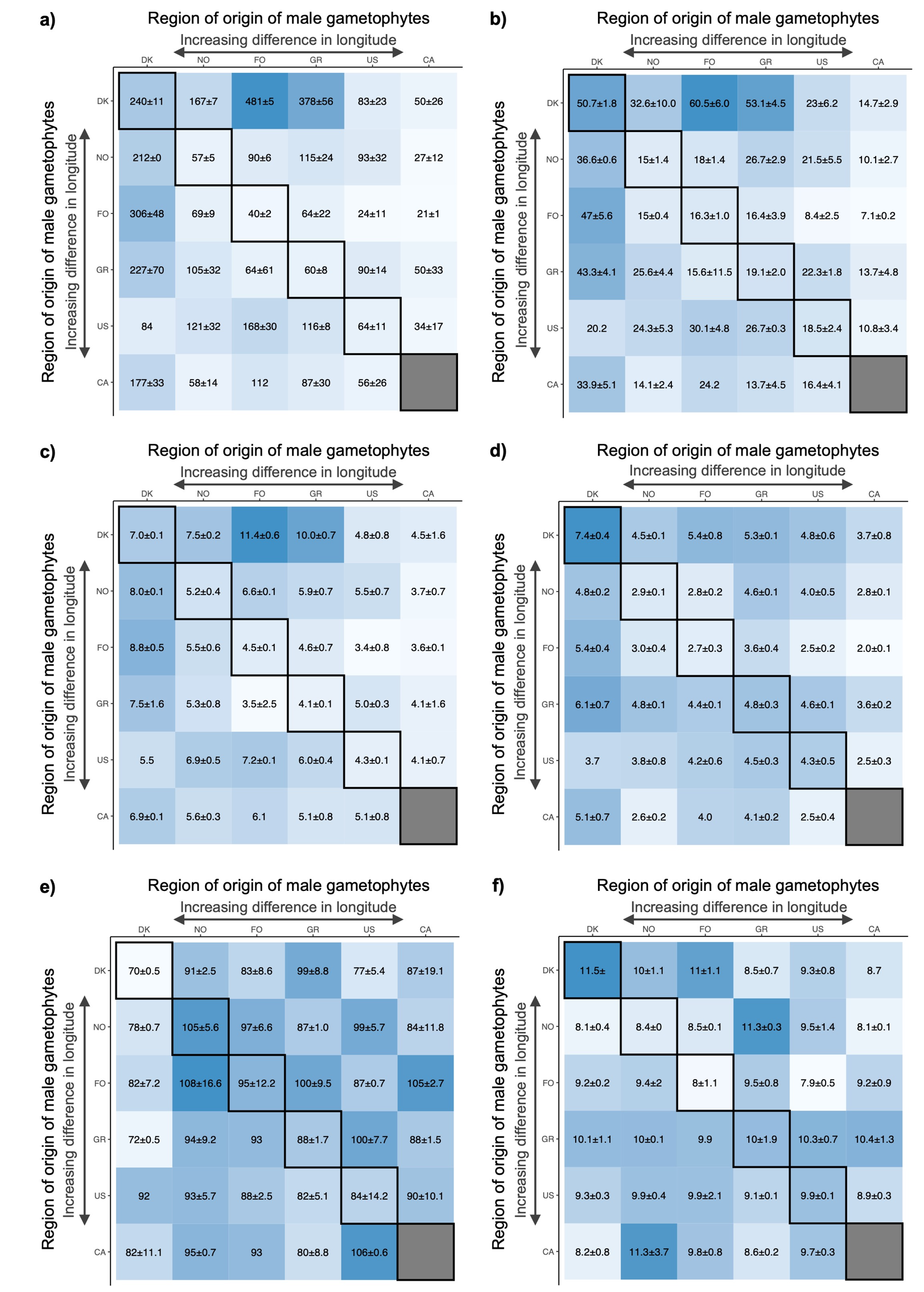


**Fig. S4** Intra- and interregional hybrid plot data heatmaps showing a) blade surface area (cm^2^) ± SE, b) blade length (cm) ± SE, c) blade maximum width (cm), d) blade length to width ratio, e) blade base angle (º) and f) dry weight percentage (%). Intraregional hybrids are found diagonally and are outlined in black. The color gradient reflects the magnitude of values
